# Effects of Changes in Metabolic Syndrome Status on Cognitive Function: A 10‐Year Study in a Middle‐Aged Population

**DOI:** 10.1002/kjm2.70138

**Published:** 2025-11-12

**Authors:** Kelly Lim, Meng‐Ni Wu, Wei‐Chia Tsao, Wei‐Hao Lin, Suh‐Hang Hank Juo, Cheng‐Sheng Chen, Hsiu‐Fen Lin

**Affiliations:** ^1^ Department of Neurology Pingtung Christian Hospital Pingtung Taiwan; ^2^ Graduate Institute of Medicine Kaohsiung Medical University Kaohsiung Taiwan; ^3^ Department of Neurology Kaohsiung Medical University Hospital Kaohsiung Taiwan; ^4^ Graduate Institute of Clinical Medicine Kaohsiung Medical University Kaohsiung Taiwan; ^5^ Department of Neurology Kaohsiung Medical University Kaohsiung Taiwan; ^6^ Ph.D. Program in Translational Medicine Kaohsiung Medical University and Academia Sinica Kaohsiung Taiwan; ^7^ Department of Medical Research China Medical University Hospital Taichung Taiwan; ^8^ Institute of Translational Medicine and new Drug Development China Medical University Taichung Taiwan; ^9^ Drug Development Center China Medical University Taichung Taiwan; ^10^ Department of Psychiatry, College of Medicine Kaohsiung Medical University Kaohsiung Taiwan; ^11^ Department of Psychiatry Kaohsiung Medical University Hospital Kaohsiung Taiwan

**Keywords:** cognition, longitudinal studies, metabolic syndrome, middle aged, sex difference

## Abstract

The long‐term cumulative impact of metabolic syndrome (MS) on cognitive decline remains uncertain. This study investigated how changes in MS status over 10 years relate to cognition and whether sex modifies this relationship. A total of 766 participants (mean baseline age: 54 years) from the Kaohsiung Atherosclerosis Longitudinal Study were enrolled. MS was defined using the modified National Cholesterol Education Program Adult Treatment Panel III criteria for Asian populations. Participants were categorized into four groups based on changes in MS status over the 10‐year follow‐up: never MS, ever MS, new MS, and persistent MS. Cognition was assessed using the Chinese version of the Montreal Cognitive Assessment (MoCA). Multivariate regression models were adjusted for age, sex, education, smoking status, physical activity, alcohol consumption, anxiety and depression. The results showed that participants with persistent MS had lower MoCA scores (*β* = −0.08, adjusted *p* = 0.020) compared to those who never had MS, with impairments primarily in the memory and language domains. This adverse effect was observed only in women (*β* = −0.12, adjusted *p* = 0.004), while no significant association was found in men (*β* = −0.03, adjusted *p* = 0.628). Individuals with nonpersistent MS (either ever or new MS) did not show significant cognitive decline compared to those who never had MS. This study demonstrates that persistent MS over a decade is linked to cognitive decline, with a more pronounced effect in women. These findings highlight the importance of early MS intervention in midlife, particularly for women, to reduce the risk of cognitive deterioration later in life.

## 1 Introduction

Metabolic syndrome (MS) comprises a constellation of vascular risk factors that collectively increase the risks of stroke, cardiovascular disease, and diabetes mellitus [1]. The vascular risk factors are also well‐established contributors to cognitive decline and dementia [2]. Numerous cohort studies have shown an association between MS and dementia [3, 4]. Few longitudinal studies assessing the cumulative effect of MS on cognitive decline in individuals without dementia have been conducted.

Given that MS encompasses modifiable cardiovascular factors, understanding the effects of longitudinal changes in MS status on cognition is of considerable clinical and public health interest. To date, few studies have directly addressed this issue. A British population‐based cohort study reported that individuals with persistent MS exhibited lower cognitive performance over a 10‐year follow‐up period than did those without MS [5]. Conversely, a Dutch population‐based cohort study, which assessed MS status twice over a 3.69‐year period, revealed no significant differences in cognition between participants who experienced different changes in MS status [6]. A prospective cohort study in Taiwan reported an increased risk of dementia among participants with nonpersistent MS (i.e., MS that changed over a 5‐year period) but no such risk among participants with persistent MS [7]. A Korean study revealed that, compared with participants who remained persistently free of MS, those with worsened, improved, or persistent MS had a significantly higher risk of dementia [8]. The inconsistencies observed across these studies may stem from differences in racial or ethnic backgrounds, follow‐up durations, and study population characteristics.

MS prevalence varies by ethnicity, age, and sex [9, 10]. The effect of MS on cognition also varies by age and sex, particularly among White populations. The impact of MS on cognition appears to be more pronounced in midlife populations than in older individuals [11, 12, 13, 14]. Additionally, sex differences have been observed, with some studies reporting a greater disadvantage for women [15] and others indicating a more pronounced effect in men [16]. Therefore, using a midlife general population cohort, this study investigated the effect of 10‐year changes in MS status on cognition in a Chinese population. Furthermore, the study evaluated whether sex‐specific differences moderate the relationship between changes in MS status and cognition outcomes.

## 2 Materials and Methods

### 2.1 Study Participants

**FIGURE 1:**
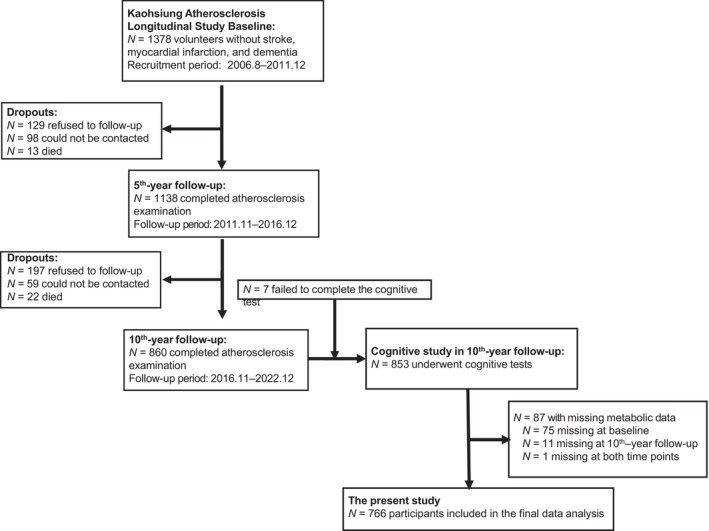
Flowchart of participant selection.

Participants were enrolled from the ongoing Kaohsiung Atherosclerosis Longitudinal Study, a cohort study that recruited patients between August 2006 and December 2011. Advertisements calling for study participants were placed at the Health Screening Center of Kaohsiung Medical University Hospital, Kaohsiung, Taiwan. Individuals aged ≥ 45 years who did not have cerebrovascular and cardiovascular diseases or dementia were eligible for inclusion. A total of 1378 participants were enrolled at baseline [17]. Follow‐up assessments, which included personal history evaluations, blood tests, and atherosclerosis examinations, were conducted at 5‐year intervals [18, 19, 20]. Of the initial cohort, 1138 participants completed the 5‐year follow‐up between November 2011 and December 2016, whereas 860 participants completed the 10‐year follow‐up between November 2016 and December 2022. Cognitive function was only assessed at the 10‐year follow‐up. A flowchart detailing participant selection is presented in Figure 1. All participants provided written informed consent, and the study protocol was approved by the Institutional Review Board of Kaohsiung Medical University Hospital.

### 2.2 Assessment of Variables

At baseline and subsequent follow‐up visits, all participants completed a self‐reported questionnaire and underwent a comprehensive metabolic status assessment. The questionnaire collected sociodemographic information, including sex, age, smoking habit, alcohol consumption, physical activity, education level, and medical history. The participants were classified as smokers if they were either current or former smokers. Alcohol consumption was categorized as ≥ 2 days per week versus < 2 days per week. Physical activity was defined by self‐reported regular exercise and categorized as high (≥ 3 times per week) or low‐to‐moderate (< 3 times per week). Education level was recorded as a continuous variable, measured in years of formal education. Blood pressure was measured using a calibrated standard sphygmomanometer (Omron; Vernon Hills, IL, USA). Two readings were obtained for each participant following a 5‐min rest period, and the average of these measurements was used for analysis. Waist circumference was measured as the abdominal circumference at the level of the anterior superior iliac spine and recorded in centimeters. Total cholesterol, high‐density lipoprotein cholesterol (HDL‐C), triglycerides (TG), and fasting glucose levels were determined from venous blood samples collected after a minimum fasting period of 8 h.

### 2.3 Definition of the MS and Changes in MS Status

MS was defined in accordance with the National Cholesterol Education Program Adult Treatment Panel III (NCEP‐ATP III) criteria, modified to account for waist circumference thresholds specific to Asian populations [21, 22]. Participants had MS if they met three or more of the following criteria: (1) elevated waist circumference (> 90 cm in men; > 80 cm in women); (2) elevated TG (≥ 150 mg/dL) or current treatment for elevated TG; (3) reduced HDL‐C (< 40 mg/dL in men; < 50 mg/dL in women) or current treatment for reduced HDL‐C; (4) elevated fasting glucose (≥ 100 mg/dL) or current treatment for elevated glucose; (5) elevated blood pressure (systolic ≥ 130 and/or diastolic ≥ 85 mmHg) or current antihypertensive treatment. MS status was assessed at baseline and at each follow‐up visit.

Changes in MS status over the 10‐year follow‐up period were classified into four groups: never MS (consistent normal status), ever MS (transition from MS to normal), new MS (transition from normal to MS), and persistent MS (consistent MS status).

### 2.4 Cognitive Function Assessment

Cognitive function was assessed in the 10th year by using the Chinese version of the Montreal Cognitive Assessment (MoCA) [23, 24]. The MoCA provides a total score of between 0 and 30, with higher scores indicating better cognitive function. Additionally, it provides subscores derived from various combination tests, including the memory index score (MIS), executive index score (EIS), language index score (LIS), visuospatial index score (VIS), attention index score (AIS), and orientation index score (OIS) [25].

The MIS is calculated by summing the numbers of words recalled across three memory tasks: free delayed recall, category‐cued recall, and multiple choice–cued recall. The values obtained in these tasks are multiplied by 3, 2, and 1, respectively, resulting in a score of between 0 and 15. The EIS is determined by summing the scores on tasks assessing executive function, including trail‐making, clock drawing, digit span, letter A tapping, serial 7 subtraction, letter fluency, and abstraction, with total scores of between 0 and 13. The VIS, ranging from 0 to 7, is computed by summing the scores of cube copy, clock drawing, and naming. The LIS is calculated as the sum of scores of naming, sentence repetition, and letter fluency, with a total score ranging from 0 to 6. The AIS is obtained by summing the scores of digit span, letter A tapping, serial 7 subtraction, sentence repetition, and words recalled in both immediate recall trials, yielding a score ranging from 0 to 18. Finally, the OIS is calculated by summing the scores of all the orientation items in the MoCA test, with a score ranging from 0 to 6.

### 2.5 Mental Health Status Assessment

Mental health status was assessed using the Chinese version of the Hospital Anxiety and Depression Scale (HADS) at the 10th‐year follow‐up, concurrent with the cognitive evaluation [26]. The self‐administered HADS includes a 7‐item anxiety subscale and a 7‐item depression subscale. Each item is scored on a four‐point scale (0–3), yielding maximum subscale scores of 21 for both anxiety and depression. Higher scores indicate greater psychological distress.

### 2.6 Statistical Analysis

Continuous variables are presented as means ± standard deviations (SDs). Categorical variables are presented as numbers (percentages). Sex‐based differences in categorical variables and continuous variables were assessed using the *χ* ^2^ test and independent *t* test, respectively. Cognitive performance across groups with different MS status changes was compared using analysis of variance (ANOVA) for univariate analysis. Multivariate regression analysis was employed to investigate the association between MS status changes and cognitive outcomes, with MoCA total and subdomain scores as dependent variables. These models were adjusted for potential confounders, including age, sex, education level, smoking status, physical activity, alcohol consumption, anxiety and depression. MS status change was included as a categorical variable using dummy coding. To explore potential sex‐specific differences, the analyses were stratified by sex, and data were reanalyzed separately for men and women. All statistical analyses were conducted using SPSS 19.0 (SPSS, Chicago, IL, USA). A two‐tailed *p* value of < 0.05 was considered statistically significant.

## 3 Results

### 3.1 Demographic Data

Of the 860 participants who completed the 10th‐year follow‐up, 7 participants did not complete the cognitive assessment, and 87 participants lacked metabolic data (75 at baseline, 11 at the 10th‐year follow‐up, and 1 at both time points). Consequently, 766 participants were included in the final analysis. No significant differences in demographic profiles were noted between the participants included in the study and those who were excluded due to missing data (Table S1). The baseline characteristics of the 766 participants are presented in Table 1. At baseline, the mean age of the participants was 54.46 ± 8.01 years, with men comprising 39.82% of the cohort. The overall prevalence of MS at baseline was 26.11%. Compared with women, men were significantly older, had attained higher education levels, and were more likely to be smokers and to consume alcohol. Regarding metabolic variables, men exhibited higher systolic and diastolic blood pressure, fasting glucose, TG, and waist circumference and lower levels of HDL‐C. Additionally, the prevalence of MS at baseline was higher among men (30.49%) than among women (23.21%).

**TABLE 1:** Baseline characteristics of study participants.

	All	Men	Women	*p* (men vs. women)
	*N* = 766	*N* = 305 (39.82%)	*N* = 461 (60.18%)	
Baseline				
Age (years)	54.46 ± 8.01	55.89 ± 8.25	53.51 ± 7.72	< 0.001*
Education (years)	13.08 ± 2.86	13.83 ± 2.45	12.58 ± 3.00	< 0.001*
Ever and current smoker	152 (19.84%)	141 (46.23%)	11 (2.39%)	< 0.001*
High physical activity	362 (47.26%)	150 (49.18%)	212 (45.99%)	0.416
Alcohol consumption	31 (4.05%)	23 (7.54%)	8 (1.74%)	< 0.001*
Systolic BP (mmHg)	117.47 ± 14.33	119.03 ± 12.50	116.42 ± 15.36	0.011*
Diastolic BP (mmHg)	73.46 ± 8.55	74.37 ± 7.86	72.85 ± 8.93	0.014*
Fasting glucose (mg/dL)	100.80 ± 20.60	104.18 ± 21.79	98.57 ± 19.47	< 0.001*
Triglycerides (mg/dL)	118.62 ± 69.22	135.15 ± 74.30	107.68 ± 63.41	< 0.001*
HDL cholesterol (mg/dL)	57.04 ± 15.28	49.58 ± 11.38	61.92 ± 15.53	< 0.001*
Waist circumference (cm)	82.90 ± 10.02	88.11 ± 8.39	79.45 ± 9.51	< 0.001*
MS (%)	200 (26.11%)	93 (30.49%)	107 (23.21%)	0.025*
High BP (%)	268 (34.99%)	119 (39.02%)	149 (32.32%)	0.067
High triglycerides (%)	194 (25.33%)	104 (34.10%)	90 (19.52%)	< 0.001*
Low HDL cholesterol (%)	150 (19.58%)	57 (18.69%)	93 (20.17%)	0.672
High fasting glucose (%)	294 (38.38%)	148 (48.52%)	146 (31.67%)	< 0.001*
High WC (%)	330 (43.08%)	119 (39.02%)	211 (45.77%)	0.062
10th‐year follow‐up				
Age (years)	65.36 ± 8.17	66.85 ± 8.37	64.37 ± 7.90	< 0.001*
Systolic BP (mmHg)	135.66 ± 17.01	137.25 ± 16.04	134.60 ± 17.56	0.038*
Diastolic BP (mmHg)	83.56 ± 9.19	86.28 ± 9.00	81.75 ± 8.87	< 0.001*
Fasting glucose (mg/dL)	103.48 ± 23.19	107.84 ± 28.71	100.62 ± 18.18	< 0.001*
Triglycerides (mg/dL)	111.44 ± 73.39	118.84 ± 77.67	106.57 ± 70.09	0.024*
HDL cholesterol (mg/dL)	59.09 ± 16.06	51.67 ± 13.98	63.96 ± 15.48	< 0.001*
Waist circumference (cm)	82.80 ± 10.19	89.12 ± 8.64	78.61 ± 8.90	< 0.001*
MS (%)	271 (35.38%)	127 (41.64%)	144 (31.24%)	0.003*
High BP (%)	547 (71.41%)	246 (80.66%)	301 (65.29%)	< 0.001*
High triglycerides (%)	140 (18.28%)	63 (20.66%)	77 (16.70%)	0.153
Low HDL cholesterol (%)	137 (17.89%)	52 (17.05%)	85 (18.44%)	0.653
High fasting glucose (%)	366 (47.78%)	168 (55.08%)	198 (42.95%)	< 0.001*
High WC (%)	339 (44.26%)	138 (45.25%)	201 (43.60%)	0.654
Anxiety	3.81 ± 3.62	3.17 ± 3.34	4.23 ± 3.75	< 0.001*
Depression	3.82 ± 3.66	3.69 ± 3.60	3.90 ± 3.70	0.447
MoCA				
Total score	25.87 ± 2.97	25.80 ± 2.97	25.92 ± 2.97	0.588
Subdomain scores				
MIS	12.42 ± 2.54	11.92 ± 2.76	12.74 ± 2.33	< 0.001*
EIS	11.62 ± 1.43	11.71 ± 1.39	11.56 ± 1.46	0.139
VIS	6.45 ± 0.92	6.59 ± 0.75	6.35 ± 1.00	< 0.001*
LIS	5.05 ± 1.06	5.07 ± 1.01	5.04 ± 1.09	0.756
AIS	16.00 ± 2.07	15.91 ± 2.20	16.06 ± 1.98	0.312
OIS	5.86 ± 0.42	5.83 ± 0.50	5.89 ± 0.35	0.064
Changes in MS status				
Never MS	443 (57.83%)	157 (51.48%)	286 (62.04%)	0.022*
Ever MS	52 (6.79%)	21 (6.89%)	31 (6.72%)	
New MS	123 (16.06%)	55 (18.03%)	68 (14.75%)	
Persistent MS	148 (19.32%)	72 (23.61%)	76 (16.49%)	

*Note*: Data are presented as mean ± SD or *n* (%). The *χ* ^2^ test was used for assessing categorical variables, and the independent *t* test was used for assessing continuous variables.

Abbreviations: AIS, attention index score; BP, blood pressure; EIS, executive index score; HDL, high‐density lipoprotein; LIS, language index score; MIS, memory index score; MoCA, Montreal Cognitive Assessment; MS, metabolic syndrome; OIS, orientation index score; VIS, visuospatial index score; WC, waist circumference.

* *p* < 0.05.

The average follow‐up duration was 10.40 ± 0.79 years. At the 10th‐year follow‐up, the mean age of the participants was 65.36 ± 8.17 years. The overall prevalence of MS at follow‐up was 35.38%, with a higher prevalence observed in men (41.64%) than in women (31.24%). Regarding cognitive function, the mean MoCA total score was 25.87 ± 2.97 across all participants, and no significant sex‐based differences were observed.

Regarding the dynamic changes in MS status over the 10‐year follow‐up period, the participants were categorized as follows: 57.83% never had MS, 6.79% had experienced MS, 16.06% had received a new diagnosis of MS, and 19.32% had persistent MS. Comparisons by sex revealed that men had a significantly higher prevalence of persistent MS and a lower prevalence of never having MS compared with women (*p* = 0.022).

### 3.2 Association Between Changes in MS Status and Cognitive Function

Table 2 presents the results of the univariate analysis examining cognitive function in relation to changes in MS status. Compared with the never MS group (MoCA score = 26.07 ± 2.93), the persistent MS group exhibited a significantly lower overall MoCA score (MoCA score = 25.23 ± 3.21; *p* = 0.018). No significant differences in overall MoCA scores were observed between the ever MS or new MS groups and the never MS group. An analysis of MoCA subdomains revealed that the persistent MS group had a significantly lower score in the MIS and LIS domains than did the never MS group (*p* = 0.026 and 0.011, respectively).

**TABLE 2:** Results of univariate analysis of MoCA total and subdomain scores by changes in MS status.

	All				Men				Women			
	Never MS	Ever MS	New MS	Persistent MS	Never MS	Ever MS	New MS	Persistent MS	Never MS	Ever MS	New MS	Persistent MS
	*N* = 443	*N* = 52	*N* = 123	*N* = 148	*N* = 157	*N* = 21	*N* = 55	*N* = 72	*N* = 286	*N* = 31	*N* = 68	*N* = 76
MoCA												
Total score	26.07 ± 2.93	26.00 ± 2.75	25.87 ± 2.83	25.23 ± 3.21	25.66 ± 3.21	25.95 ± 2.62	26.35 ± 2.28	25.64 ± 2.98	26.29 ± 2.73	26.03 ± 2.88	25.49 ± 3.17	24.84 ± 3.38
*p*		1.000	1.000	0.018*		1.000	0.840	1.000		1.000	0.252	< 0.001*
Subdomain score												
MIS	12.60 ± 2.54	12.60 ± 2.20	12.26 ± 2.43	11.92 ± 2.70	11.82 ± 2.91	12.62 ± 2.09	12.51 ± 2.04	11.49 ± 2.99	13.03 ± 2.20	12.58 ± 2.31	12.06 ± 2.70	12.33 ± 2.32
*p*		1.000	1.000	0.026*		1.000	0.665	1.000		1.000	0.011*	0.108
EIS	11.63 ± 1.39	11.67 ± 1.41	11.71 ± 1.49	11.49 ± 1.51	11.61 ± 1.41	11.57 ± 1.29	12.02 ± 1.23	11.75 ± 1.47	11.64 ± 1.38	11.74 ± 1.51	11.46 ± 1.64	11.25 ± 1.52
*p*		1.000	1.000	1.000		1.000	0.347	1.000		1.000	1.000	0.229
VIS	6.48 ± 0.89	6.33 ± 1.06	6.56 ± 0.80	6.31 ± 1.02	6.57 ± 0.79	6.57 ± 0.81	6.73 ± 0.62	6.54 ± 0.75	6.42 ± 0.936	6.16 ± 1.19	6.43 ± 0.90	6.09 ± 1.19
*p*		1.000	1.000	0.343		1.000	1.000	1.000		0.990	1.000	0.062
LIS	5.12 ± 0.98	5.00 ± 1.22	5.10 ± 1.08	4.81 ± 1.20	5.04 ± 0.97	5.00 ± 1.10	5.16 ± 1.05	5.07 ± 1.07	5.17 ± 0.98	5.00 ± 1.32	5.04 ± 1.11	4.57 ± 1.27
*p*		1.000	1.000	0.011*		1.000	1.000	1.000		1.000	1.000	< 0.001*
AIS	16.09 ± 2.08	15.92 ± 2.03	15.99 ± 2.13	15.76 ± 2.02	15.80 ± 2.41	15.14 ± 2.01	16.27 ± 1.81	16.08 ± 1.98	16.26 ± 1.86	16.45 ± 1.90	15.76 ± 2.34	15.45 ± 2.02
*p*		1.000	1.000	0.517		1.000	1.000	1.000		1.000	0.388	0.009*
OIS	5.87 ± 0.40	5.90 ± 0.30	5.89 ± 0.40	5.80 ± 0.52	5.85 ± 0.46	5.86 ± 0.36	5.87 ± 0.39	5.72 ± 0.66	5.88 ± 0.36	5.94 ± 0.25	5.90 ± 0.39	5.88 ± 0.33
*p*		1.000	1.000	0.534		1.000	1.000	0.391		1.000	1.000	1.000

*Note*: Data are presented as mean ± SD.

Abbreviations: AIS, attention index score; EIS, executive index score; LIS, language index score; MIS, memory index score; MoCA, Montreal Cognitive Assessment; MS, metabolic syndrome; OIS, orientation index score; VIS, visuospatial index score.

* *p* < 0.05.

Table 3 presents the results of the multivariate analysis assessing cognitive function in relation to changes in MS status. After adjustment for age, sex, education level, smoking status, physical activity, alcohol consumption, anxiety and depression, the persistent MS group demonstrated a significantly lower overall MoCA scores than did the never MS group (*β* = −0.08; adjusted *p* = 0.020). No significant difference in overall MoCA score was observed between the ever MS (*β* = 0.01; adjusted *p* = 0.791) or new MS (*β* = −0.02; adjusted *p* = 0.451) groups and the never MS group. Further evaluation of cognitive subdomains indicated that the MIS and LIS domains were the most significantly affected in the persistent MS and never MS groups, with adjusted *p* values of 0.040 and 0.005, respectively.

**TABLE 3:** Results of multivariate analysis of MoCA total scores by changes in MS status, after adjustment for covariates.

	All^a^			Men^b^			Women^b^		
	*β*	95% CI	*p*	*β*	95% CI	*p*	*β*	95% CI	*p*
Ever MS/Never MS	0.01	−0.65 to 0.85	0.791	0.01	−1.09 to 1.38	0.820	−0.01	−1.07 to 0.83	0.802
New MS/Never MS	−0.02	−0.71 to 0.32	0.451	0.02	−0.67 to 0.95	0.733	−0.06	−1.17 to 0.16	0.138
Persistent MS/Never MS	−0.08	−1.05 to −0.09	0.020*	−0.03	−0.92 to 0.56	0.628	−0.12	−1.61 to −0.32	0.004*
Baseline age	−0.26	−0.12 to −0.07	< 0.001*	−0.37	−0.17 to −0.10	< 0.001*	−0.15	−0.09 to −0.03	< 0.001*
Education years	0.41	0.36 to 0.49	< 0.001*	0.31	0.25 to 0.49	< 0.001*	0.46	0.38 to 0.53	< 0.001*
Smoking status	0.12	0.37 to 1.46	0.001*	0.12	0.10 to 1.32	0.022*	0.07	−0.22 to 2.81	0.093
High physical activity	−0.01	−0.42 to 0.33	0.833	−0.00	−0.62 to 0.61	0.991	−0.02	−0.62 to 0.34	0.571
Alcohol consumption	−0.03	−1.37 to 0.50	0.365	0.02	−0.91 to 1.37	0.691	−0.11	−4.20 to −0.69	0.006*
Anxiety	0.02	−0.06 to 0.09	0.674	0.01	−0.11 to 0.13	0.895	0.03	−0.06 to 0.11	0.552
Depression	−0.05	−0.11 to 0.03	0.252	−0.06	−0.16 to 0.06	0.392	−0.04	−0.12 to 0.05	0.430
Sex	−0.13	−0.24 to −0.32	< 0.001*	—	—	—	—	—	—

Abbreviations: MoCA, Montreal cognitive assessment; MS, metabolic syndrome.

* *p* < 0.05.

^**a**^ Adjusted for age, sex, education years, smoking status, physical activity, alcohol consumption, anxiety and depression.

^**b**^ Adjusted for age, education years, smoking status, physical activity, alcohol consumption, anxiety and depression.

### 3.3 Sex‐Specific Effects of Changes in MS Status on Cognitive Function

To assess potential sex‐specific effects, we conducted separate analyses evaluating the association between changes in MS status and cognitive function in men and women. Among women, the persistent MS group exhibited significantly lower overall MoCA scores (24.84 ± 3.38) than did the never MS group (26.29 ± 2.73; *p* < 0.001; Table 2). This association remained significant even after adjustment for confounding factors, with women in the persistent MS group still exhibiting significantly lower overall MoCA scores than those in the never MS group (*β* = −0.12; adjusted *p* = 0.004; Table 3). Conversely, men did not exhibit significant differences in overall MoCA scores across the MS status groups, either in univariate or multivariate analyses.

## 4 Discussion

This longitudinal follow‐up study demonstrated that 10‐year changes in MS status can significantly influence cognitive function in midlife individuals without dementia. Specifically, individuals with persistent MS exhibited significantly lower scores in cognitive function assessments; those with nonpersistent MS status (including both the ever MS and new MS groups) did not exhibit the same lower scores. Notably, this effect was evident only in women and not in men. This is the first study to demonstrate the effect of longitudinal changes in MS status on cognition in an Asian population without dementia. These findings underscore the importance of considering sex‐specific effects.

MS encompasses several modifiable cardiovascular risk factors that collectively contribute to cognitive decline. Researchers should consider the cumulative effects of MS and not only examine MS at a single time point. Despite the importance of this approach, only a few studies have explored this relationship, and current findings remain inconsistent. The previous relevant studies and ours were summarized in Table 4. In the Whitehall II British cohort study, individuals with persistent MS exhibited lower cognitive function compared with those who never had MS. This result is based on MS assessments conducted three times over a 10‐year follow‐up [5]. By contrast, the Lifelines Cohort Study conducted in the Netherlands, which evaluated MS status at two time points over a 3.69‐year interval, reported no significant differences in cognition among participants with varying MS status changes [6]. When using dementia risk as an endpoint, findings from a Taiwanese prospective cohort study that assessed MS status at 5‐year intervals indicated that a higher dementia risk was observed only in the nonpersistent MS group and that no such association was noted in those with persistent MS [7]. Conversely, a Korean cohort study that analyzed MS status at 2‐year intervals reported that individuals with persistent or newly developed MS exhibited a higher risk of dementia compared with those sustained free of MS [8]. Consistent with the Whitehall II British cohort study findings, this study demonstrated that persistent MS is associated with poorer cognition in an Asian population without dementia. Supporting these results, Cho et al. used an MS exposure‐weighted score and reported that greater cumulative exposure to MS was associated with a higher risk of all‐cause dementia [27]. Similarly, a UK Biobank study revealed that MS was associated with increased dementia risk, particularly in follow‐up periods exceeding 10 years [4]. These findings reinforce our results, highlighting that once an individual develops MS, the cumulative negative effects, if left unaddressed, can significantly impair cognition.

**TABLE 4:** Summary of longitudinal studies examining the association between changes in MS status and cognitive outcomes in non‐demented populations.

Author, year (study)	Population (country)	Sample size & baseline age	Study design & follow‐up	Cognitive measures	Outcome	Sex analysis	Main findings
Fan et al., 2017 (Taiwan NHIRD) [7]	Taiwan	*N* = 3458; 40–80 years	Prospective cohort; 10 years	—	Dementia risk	No	Non‐persistent MS ↑ dementia risk
Lee et al., 2020 (KNHI) [8]	Korea	*N* = 4,106,590; mean 55.8 years	Population‐based cohort; 4.9 years	—	Dementia risk	No	Worsened, improved, and persistent MS all ↑ dementia risk vs. persistent MS‐free
Cho et al., 2021 (KNHI) [27]	Korea	*N* = 1,492,776; ≥ 45 years	Population‐based cohort; 4 years	—	Dementia risk	No	Greater cumulative MS exposure ↑ dementia risk
Akbaraly et al., 2010 (Whitehall II) [5]	UK civil servants	*N* = 4150; 35–55 years	Prospective cohort; 10 years	Six‐test cognitive battery	Cognitive decline	No	Persistent MS ↓cognition vs. never MS; No difference between nonpersistent MS and never MS
Frentz et al., 2024 (Lifelines) [6]	Netherlands	*N* = 14,609; mean 60.8 years	Prospective cohort; 3.7 years	Cogstate Brief Battery	Cognitive decline	No	No significant differences across MS status changes
Lin et al., present study (KALS)	Taiwan	*N* = 766; mean 54.5 years	Prospective cohort; 10 years	Montreal Cognitive Assessment (MoCA)	Cognitive decline	Yes	Persistent MS ↓cognition vs. never MS; effect observed only in women

Abbreviation: MS, metabolic syndrome.

Although the prevalence of MS increases with age [10], its effect on cognition varies across life stages. Midlife MS is considered a stronger predictor of cognitive decline than late‐life MS. The Whitehall II study demonstrated that the presence of MS components before the age of 60 was associated with a higher dementia risk (hazard ratio 1.23), whereas this association weakened after the age of 70 (hazard ratio 1.10) [14]. Similarly, a Greek population study found that middle‐aged individuals with MS performed worse on logical Memory‐delayed recall (*β* = −0.70 words, *p* = 2.65e^−5^) compared with those without MS, while no significant differences were observed in older participants (*β* = 0.35, *p* = 0.34) [13]. In a large study of diverse middle‐aged and older Hispanics/Latinos, MS was associated with lower neurocognitive function in midlife (45–64 years), but this association was less pronounced in older adults (≥ 65 years) [11]. The mechanisms underlying the association between MS and dementia risk may involve chronic inflammation, oxidative stress, and cerebrovascular damage, all of which contribute to neurodegeneration and impaired cognition [28]. Compared with midlife MS, late‐life MS may exert a less pronounced cumulative effect on the brain due to a shorter duration of exposure. Our research group focused on a middle‐aged Chinese population, and our findings reinforce the importance of effective midlife MS management in mitigating its long‐term effect on cognitive health.

Studies examining the relationship between MS and cognition have yielded inconsistent findings regarding sex differences, with some studies indicating a greater cognitive disadvantage in women [15, 29] and others suggesting a greater disadvantage in men [16]. In this study, MS status changes only affected cognition among women; no significant effect occurred among men. Findings from the Study of Women's Health Across the Nation (SWAN) demonstrated that middle‐aged women with MS undergoing menopause experienced greater cognitive decline [30]. Consistent with the SWAN study, the mean age of participants in our cohort at baseline was 54 years, aligning with the median age of natural menopause for women. A key factor contributing to this sex disparity is the role of estrogen, which exerts neuroprotective effects by supporting cerebral blood flow, synaptic plasticity, and glucose metabolism in the brain. However, as women transition through menopause, declining estrogen levels increase their vulnerability to metabolic dysregulation and cognitive impairment [31, 32]. By contrast, men do not experience a similar abrupt hormonal shift, which may explain the less pronounced cognitive effects of MS in middle‐aged men. Additionally, women undergoing menopausal transition are at a higher cardiovascular risk than men [33, 34]. The present findings underscore the need for early metabolic health interventions to mitigate the risk of future cognitive decline, particularly in midlife women.

This study demonstrated that persistent MS had a particularly pronounced effect on memory and language function. Tsai et al. identified hyperglycemia, high blood pressure, and abdominal obesity as contributing factors to frontal lobe dysfunction [35]. Additionally, hyperglycemia linked to attention and memory impairment in a cohort of young to middle‐aged individuals without dementia [36]. Sex differences in the cognitive effects of MS have also been observed. Ekblad et al. reported that higher insulin resistance linked to poorer verbal fluency in young to middle‐aged women [15]. The mechanisms by which MS affects cognition may disrupt neural pathways essential for memory and language processing in the temporal and frontal lobes [37]. In this study, the cognitive impairments primarily observed in memory and language domains further support these findings.

Our study has both strengths and limitations. A key strength is the large and relatively healthy study population, which excluded individuals with cardiovascular disease and dementia, allowing for a clearer assessment of MS and its cognitive effects over a 10‐year period. This long follow‐up enables a more comprehensive examination of the effect of persistent MS on cognition. Furthermore, the use of the Chinese version of the MoCA ensured a culturally appropriate and validated measure of cognitive function in the study population. Nevertheless, several limitations should be acknowledged. Cognition was assessed only at the 10th‐year follow‐up, which precluded an analysis of cognitive trajectories over time. Apolipoprotein E (APOE) ε4 genotype information was not collected in the study; the absence of genetic data precluded us from assessing potential gene–environment interactions. Additionally, the study sample was drawn from a single population in Taiwan, limiting the generalizability of the findings to other ethnic groups.

In conclusion, the study findings indicate that persistent MS is associated with cognitive deterioration in middle‐aged individuals without dementia. This finding underscores the importance of modifying cardiometabolic risk factors during midlife to mitigate cognitive decline in later life, particularly in middle‐aged women.

## Conflicts of Interest

The authors declare no conflicts of interest.

## Supporting information


**Table S1:** Demographic characteristics of included participants and those who dropped out.

## Data Availability

The data that support the findings of this study are available on request from the corresponding author. The data are not publicly available due to privacy or ethical restrictions.
