# Supplementary material for: Effects of Changes in Metabolic Syndrome Status on Cognitive Function: A 10‐Year Study in a Middle‐Aged Population
Source: Kaohsiung J Med Sci. 2025 Nov 12;42(5):e70138. doi: 10.1002/kjm2.70138 (PMC13182602; doi:10.1002/kjm2.70138)
Supplement: Supplementary file 1 — Table S1: Demographic characteristics of included participants and those who dropped out. [file KJM2-42-e70138-s001.docx]

Supplementary Table S1. Demographic characteristics of included participants and those who dropped out.

|  | **Participants** | **Dropped out** | ***p*** |
| --- | --- | --- | --- |
|  | *N* = 766 | *N* = 94 |  |
| **Baseline** |  |  |  |
| Age (years) | 54.46±8.01 | 55.21±8.26 | 0.392 |
| Sex (men) | 305 (39.82%) | 32 (34%) | 0.279 |
| Education (years) | 13.08±2.86 | 12.54±3.48 | 0.096 |
| Ever and current smoker | 152 (19.84%) | 15 (15.96%) | 0.369 |
| Systolic BP, missing | 0 (0%) | 2 (2.13%) |  |
| Diastolic BP, missing | 0 (0%) | 2 (2.13%) |  |
| Fasting glucose, missing | 0 (0%) | 57 (60.64%) |  |
| Triglycerides, missing | 0 (0%) | 52 (55.32%) |  |
| HDL cholesterol, missing | 0 (0%) | 71 (75.53%) |  |
| Waist circumference, missing | 0 (0%) | 23 (24.47%) |  |
| **10^th^-year follow-up** |  |  |  |
| Systolic BP, missing | 0 (0%) | 10 (10.64%) |  |
| Diastolic BP, missing | 0 (0%) | 10 (10.64%) |  |
| Fasting glucose, missing | 0 (0%) | 2 (2.13%) |  |
| Triglycerides, missing | 0 (0%) | 2 (2.13%) |  |
| HDL cholesterol, missing | 0 (0%) | 2 (2.13%) |  |
| Waist circumference, missing | 0 (0%) | 0 (0%) |  |

Data are presented as mean ± SD or n (%).

Abbreviations: HDL: high-density lipoprotein; BP: blood pressure.

The chi-square test was used for assessing categorical variables, and the independent *t* test was used for assessing continuous variables.
